# Incidental Discovery of Nonrotation in a Patient With Nonspecific Abdominal Pain: A Surgical Diagnostic Dilemma

**DOI:** 10.7759/cureus.29153

**Published:** 2022-09-14

**Authors:** Joseph Genualdi, Max Murray-Ramcharan, Francisco Matos, Alexius Ramcharan

**Affiliations:** 1 General Surgery, Columbia University Vagelos College of Physicians and Surgeons, New York City, USA; 2 General Surgery, NYC Health + Hospitals/Harlem, New York City, USA

**Keywords:** non specific abdominal pain, differential diagnoses, incidental radiological finding, intestinal nonrotation, adult intestinal malrotation

## Abstract

Intestinal nonrotation is a subtype of malrotation occurring when the midgut fails to rotate before returning to the peritoneal cavity between weeks 8-10 of development. Though sometimes presenting as volvulus during the neonatal period, a subset of patients remains asymptomatic and are identified incidentally as adults. When patients with intestinal nonrotation present with abdominal symptoms, there exists a diagnostic dilemma for the treating surgeon. We present the case of a patient who presented with acute abdominal pain and vomiting, with radiographic findings of intestinal nonrotation and no other acute pathology. Symptoms spontaneously resolved with conservative management for likely etiology of viral gastroenteritis. At the one-month follow-up, the patient had no residual or recurrent symptoms, with no further interventions planned.

## Introduction

During normal embryologic development, the midgut herniates through the umbilical ring where it develops outside of the peritoneum around week six. It continues to rotate 270 degrees counterclockwise around the axis of the superior mesenteric artery around the eighth week before returning through the umbilical ring to the abdominal cavity around week 10 [1]. Intestinal nonrotation represents a subset of patients that fall within the greater congenital abnormality umbrella known as intestinal malrotation. During malrotation, the midgut can fail to rotate, incompletely rotate, rotate in a clockwise direction, or the mesentery can become fixed in an anomalous position. In each of the cases, a fibrous band can form from the cecum to the retroperitoneum, leading to acute complications such as small bowl obstruction or volvulus often within the first week after birth [2]. When symptomatic, these conditions represent an acute surgical emergency in the pediatric population. This differs from intestinal nonrotation in adults, which is the failure of normal embryologic rotation, without any evidence of acute pathology. This usually remains undetected until adulthood or upon postmortem analysis. In contrast to the symptomatic pediatric patient, the role of aggressive surgical management as with the Ladd's procedure or other interventions remains unclear for asymptomatic patients with nonrotation in adulthood.

## Case presentation

We present a 76-year-old woman with a past medical history of stage one breast cancer managed with right mastectomy, and further surgical history of laparoscopic umbilical hernia repair and lysis of adhesions eight years prior. The patient was seen in the emergency department for a two-day history of abdominal pain, nausea, non-bloody non-bilious emesis, and non-bloody non-mucoid diarrhea. Symptoms began acutely and she denied having similar symptoms in the past.

On examination, she was afebrile, and her vitals were stable. She endorsed that she has been having diarrhea more than five time a day for the past two days and was passing flatus. The patient’s abdomen was soft, non-distended, and exhibited generalized moderate tenderness without evidence of peritonitis. The remainder of her exam was unremarkable as were her lipase and white blood cell count.

**Figure 1:**
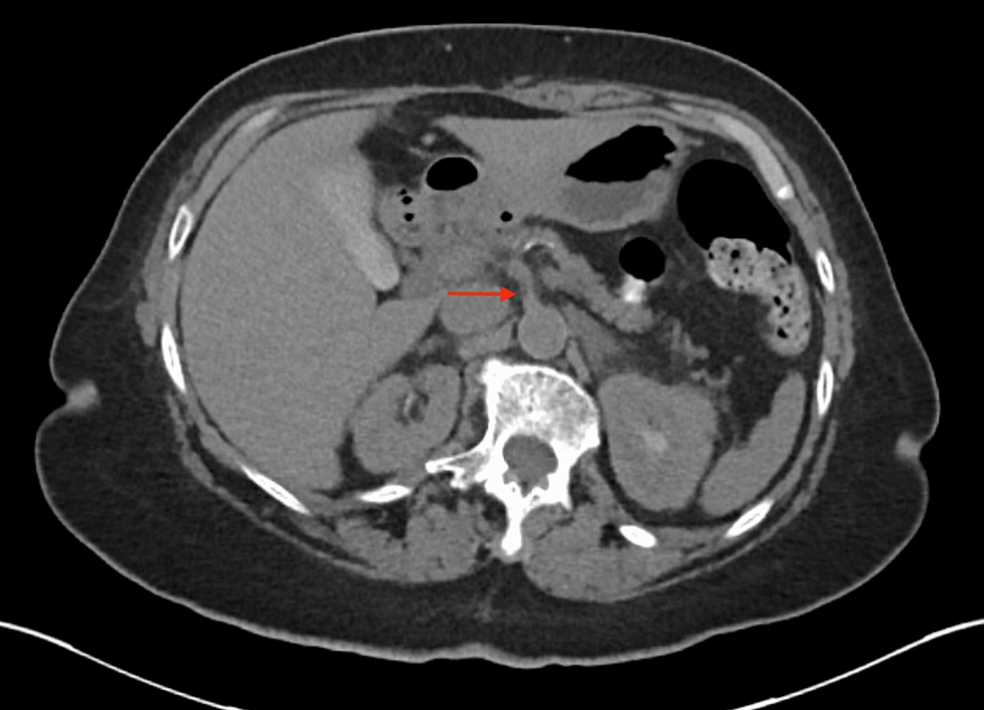
Axial CT abdomen showing the origin of the SMA (red arrow) SMA - superior mesenteric artery

**Figure 2:**
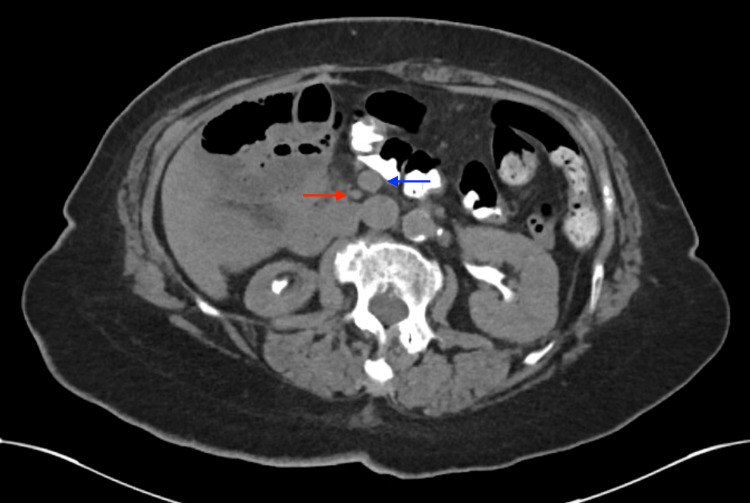
Axial CT abdomen showing the SMV (blue arrow) to the left of the SMA (red arrow), which is pathognomonic for intestinal nonrotation SMV - superior mesenteric vein; SMA - superior mesenteric artery

**Figure 3:**
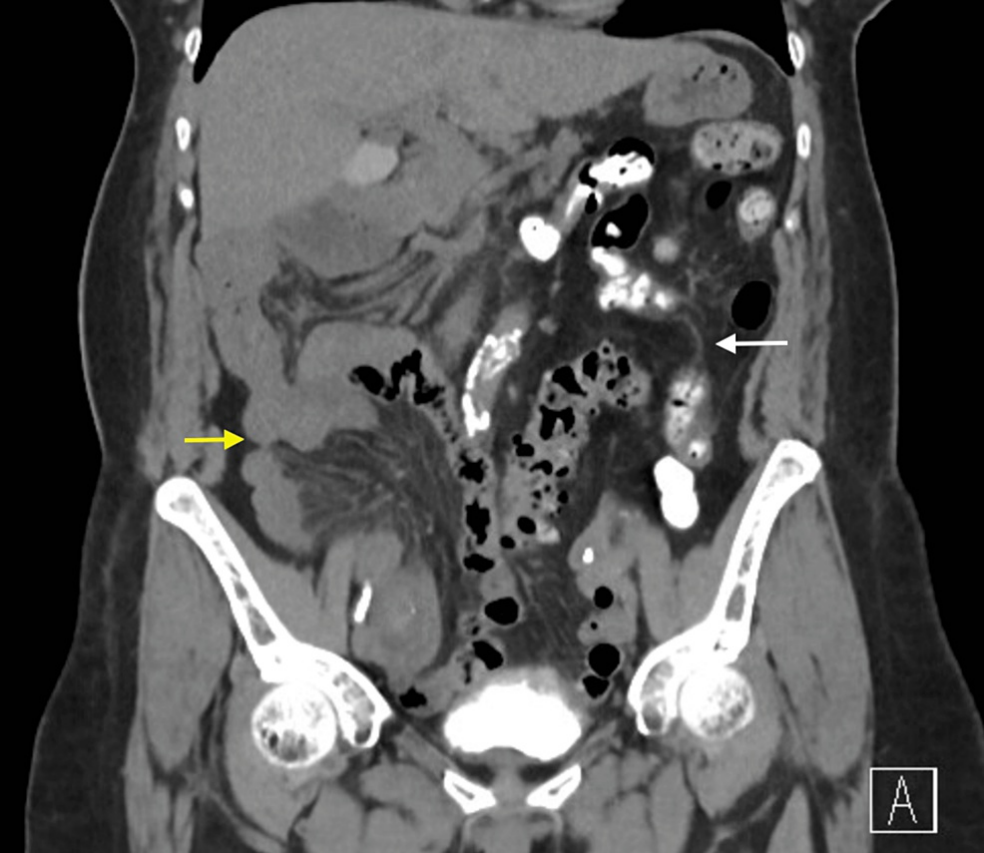
Coronal CT abdomen showing small intestine (yellow arrow) predominantly on the right side and the colon (white arrow) predominantly on the left side

A CT of the abdomen and pelvis was initially performed with intravenous contrast only and demonstrated dilated small bowl loops all gathered on the right with a fluid-filled dilated colon mostly on the left hemiabdomen. This study was repeated with oral contrast, which ruled out small bowel obstruction as an etiology. Also noted was the superior mesentery vein to the left of the superior mesenteric artery, the small intestine in the right hemiabdomen, and the colon on the left side, confirming intestinal non-rotation (Figures 1, 2, 3).

The patient was admitted to the hospital due to her inability to tolerate per oral, and was managed conservatively with fluid resuscitation, anti-emetics, and gradual diet advancement. Surgical intervention was not warranted because acute surgical emergency had not been identified and the intestinal nonrotation, although abnormal, was not causing her any obstructive symptoms. On hospital day two, she was discharged after complete resolution of symptoms with a working diagnosis of viral gastroenteritis.

One month after discharge, the patient remained asymptomatic without recurrent symptoms. She was educated about her incidental findings and informed to relay this message to treating physicians in the case of future abdominal symptoms.

## Discussion

In pediatric patients, symptomatic malrotation is commonly thought to occur in 1:6,000 live births. The exact prevalence of asymptomatic nonrotation in adults remains unclear, though some studies utilizing CT colonography databases have estimated a prevalence of malrotation of up to 0.2%, and as a subset of this population, the prevalence of intestinal nonrotation is likely much lower [3]. A review of the literature failed to show any large studies to accurately determine prevalence.

Pathognomonic radiologic findings of intestinal nonrotation include identification of the superior mesentery vein to the left of the superior mesenteric artery, a right-sided small intestine, and left-sided large bowel. The Stinger classification of intestinal malrotation (Table 1) can further be used to define nonrotation [4,5].

**Table 1:** Stringer’s classification of intestinal malrotation SMA – superior mesenteric artery

Type	Embryonic Developmental Stage		Definition
I (nonrotation)	<6^th^ week		Nonrotation of colon and duodenum
II (duodenal malrotation)	6^th^-10^th^ week	A	Duodenal nonrotation and normal colonic rotation (often results in Ladd band formation)
		B	Reverse rotation of duodenum (anterior to SMA) and colon (posterior to SMA)
		C	Reverse rotation of duodenum only (small bowel entrapment)
III (duodenal and cecal malrotation)	>10^th^ week	A	Duodenum to the right of midline, high cecum (predisposed to volvulus)
		B	Normal rotation of duodenum, incomplete fixation of hepatic flexure (often with Ladd bands)
		C	Incomplete attachment of cecum (mobile cecum)
		D	Paraduodenal hernias near ligament of Treitz due to variable fixation (pseudo-malrotation)

The case described represents type I: nonrotation of the colon and duodenum. Nonrotation is associated with significantly fewer complications than the other classes of malrotation and is oftentimes discovered incidentally during imaging or academic cadaveric dissection [6,7]. This abnormality is considered benign given the late age at which it is discovered and the paucity of symptoms or complications.

There have, however, been some reports of previously asymptomatic adults with intestinal nonrotation experiencing volvulus at up to 70 years of age [8,9]. Similarly, there are cases of patients being asymptomatic until obstruction at advanced ages [10]. It is unclear what triggers these acute complications after multiple decades of life. Additionally, there have been reports of co-occurring anatomic abnormalities, such as the presence of a middle mesentery artery, though it is unclear if these abnormalities are related or purely coincidental [11].

The difficulty in recognition and diagnosis of some conditions due to the unique anatomy has also been noted. Several reports have noted delays in the recognition of appendicitis leading to complications due the anatomical variation [12-14]. As in the cases of delayed volvulus and obstruction at an advanced age, it is unclear if these complications are influenced by intestinal nonrotation or if they are purely coincidental.

In the case presented in this report, it was ultimately decided that surgical intervention was not warranted given the lack of acute pathology noted radiographically, as well as the resolution of symptoms with conservative management. Some have advocated for a more aggressive surgical approach to correct nonrotation via the Ladd’s procedure, even in patients only experiencing occasional mild symptoms [15].

There have been few retrospective analyses published describing outcomes of surgical correction of nonrotation and often propose an aggressive approach to management to avoid catastrophic complications such as bowel strangulation or ischemia. However, surgical complication rates remain high in this population. In one review of 12 patients who underwent surgery for asymptomatic nonrotation as adults, three had post-operative complications including ileus, delayed gastric emptying, and wound infection, two more experienced nonrotation recurrence, and one required re-operation [16]. The authors of this report recommend employing a shared decision model involving a discussion of rare but potentially harmful complications if surgery is not performed, and the risks of surgery including the high likelihood of postoperative complication. Patients' willingness to undergo corrective surgery will likely vary greatly depending on symptomatology to date and age and life expectancy at the time of diagnosis. Given the rarity of nonrotation patients presenting later in life with vague symptoms, and thus the lack of randomized control trials, it is unclear what benefit might be achieved to surgical correction if asymptomatic to date. 

## Conclusions

This case describes Intestinal nonrotation (Type I malrotation in Stinger’s classification) associated with nonrelated abdominal symptoms, successfully managed without operative intervention. The few reports of nonrotation in the literature have conflicting views with regards to management, with debate between conservative management versus more aggressive surgical management, with trends favoring the former. Larger studies are needed to determine true clinical relevance of this entity and to define optimal management.
